# Optical inhibition of larval zebrafish behaviour with anion channelrhodopsins

**DOI:** 10.1186/s12915-017-0430-2

**Published:** 2017-11-03

**Authors:** Gadisti Aisha Mohamed, Ruey-Kuang Cheng, Joses Ho, Seetha Krishnan, Farhan Mohammad, Adam Claridge-Chang, Suresh Jesuthasan

**Affiliations:** 1 Lee Kong Chian School of Medicine, Nanyang Technological University, Singapore, Singapore; 2 Institute of Molecular and Cell Biology, Singapore, Singapore; 3 NUS Graduate School for Integrative Sciences and Engineering, National University of Singapore, Singapore, Singapore; 4 Duke-NUS Medical School, Singapore, Singapore

**Keywords:** Optogenetics, Zebrafish, Chloride pump, Behaviour, Neural circuit

## Abstract

**Background:**

Optical silencing of activity provides a way to test the necessity of neurons in behaviour. Two light-gated anion channels, GtACR1 and GtACR2, have recently been shown to potently inhibit activity in cultured mammalian neurons and in *Drosophila*. Here, we test the usefulness of these channels in larval zebrafish, using spontaneous coiling behaviour as the assay.

**Results:**

When the GtACRs were expressed in spinal neurons of embryonic zebrafish and actuated with blue or green light, spontaneous movement was inhibited. In GtACR1-expressing fish, only 3 μW/mm^2^ of light was sufficient to have an effect; GtACR2, which is poorly trafficked, required slightly stronger illumination. No inhibition was seen in non-expressing siblings. After light offset, the movement of GtACR-expressing fish increased, which suggested that termination of light-induced neural inhibition may lead to activation. Consistent with this, two-photon imaging of spinal neurons showed that blue light inhibited spontaneous activity in spinal neurons of GtACR1-expressing fish, and that the level of intracellular calcium increased following light offset.

**Conclusions:**

These results show that GtACR1 and GtACR2 can be used to optically inhibit neurons in larval zebrafish with high efficiency. The activity elicited at light offset needs to be taken into consideration in experimental design, although this property can provide insight into the effects of transiently stimulating a circuit.

**Electronic supplementary material:**

The online version of this article (doi:10.1186/s12915-017-0430-2) contains supplementary material, which is available to authorized users.

## Background

One approach to understanding the role of specific neurons in a given behaviour is to experimentally alter their activity. A precise means of doing this is by using light-gated channels [1–4]. Optogenetic activators such as channelrhodopsin-2 [5], ChIEF [6] and CsChrimson [7] can reversibly depolarize membrane potential with millisecond resolution and have been widely used in a range of organisms [8–11]. For numerous different behavioural functions, these tools have enabled the determination of neuronal sufficiency. However, establishing whether neurons are normally involved requires additional experiments. Optical or electrical recording can determine which activity patterns are correlated with behaviour. However, for the crucially important loss-of-function experiment, effective tools for neuronal inhibition are required.

A number of different optogenetic inhibitors have been developed. The first generation of these tools include light-actuated pumps like halorhodopsin [12, 13], which facilitates light-dependent chloride entry, and archaerhodopsin [14, 15], a proton pump that is also hyperpolarizing. A limitation of these molecules is their low conductance and the high levels of expression and illumination that are required for effective silencing. For example, the archaerhodopsin derivative Archer1 requires 3 mW/mm^2^ of light to inhibit action potentials in cultured mammalian neurons [16], while halorhodopsin requires ~20 mW/mm^2^ to inhibit activity in zebrafish neurons [17]. A second generation of optogenetic inhibitors includes genetically modified channelrhodopsins such as ChloC [18] and iC1C2 [19], which hyperpolarize neurons by light-gated conductance of chloride. Both require similar levels of illumination, although an improved version of ChloC, iChloC, requires 10 times less light [20]. More recently, naturally evolved anion-conducting channels from the alga *Guillardia theta* were shown to silence neurons at very low light levels, i.e. in the range of microwatts per square millimetre [21]. *Drosophila* experiments have confirmed that these optogenetic tools are potent inhibitors in vivo [22]. Here, we ask whether *Guillardia theta* anion channelrhodopsins (GtACR1 and GtACR2) are effective inhibitors of neural activity in the larval zebrafish, a genetically tractable vertebrate.

## Methods

The experiments here were carried out in accordance with guidelines approved by the Institutional Animal Care and Use Committee of Biopolis, Singapore.

### Generation of GtACR1 and GtACR2 transgenic zebrafish lines

Sequences encoding GtACR1 and GtACR2 fused to eYFP [22] were placed downstream of the upstream activating sequence (UAS) using Gateway cloning (Thermo Fisher Scientific). The resulting construct was cloned into a plasmid containing Tol2 sequences to facilitate integration into the zebrafish genome [23, 24]. The constructs (33 ng/μl) were then injected into *nacre* ^*-/-*^ eggs at the one-cell stage, along with *elavl3:Gal4* DNA (15 ng/μl, lacking Tol2 sequences) to induce GtACR expression, and Tol2 mRNA (33 ng/μl) to facilitate genomic integration. Embryos were screened after 24–36 hs for eYFP expression. Healthy embryos with eYFP expression were grown to adulthood. At 2 months, fish were fin-clipped and PCR-screened with GtACR-specific primers (GtACR1 forward 5’-CACCGTGTTCGGCATCAC-3’, GtACR1 reverse 5’-GCCACCACCATCTCGAAG-3’; GtACR2 forward 5’-ATTACCGCTACCATCTCCCC-3’, GtACR2 reverse 5’-TGGTGAACACCACGCTGTAT-3’) to test for the presence of transgene. This led to the generation of two transgenic lines, *Tg(UAS:GtACR1-eYFP)sq211* and *Tg(UAS:GtACR2:eYFP)sq212*.

### Zebrafish lines

Transgenic lines used in this study are *elavl3:GCaMP6f* [25], *TgBAC(gng8:GAL4)* ^*c416*^ [26], *Et(-0.6hsp70l:Gal4-VP16)s1020t* [27], *Et(-0.6hsp70l:Gal4-VP16)s1011t* [27] and *UAS:NpHR-mCherry* [17]. For brevity, the enhancer trap lines are referred to as *GAL4s1020t* and *GAL4s1011t* in the text and figures.

### Confocal imaging

Twenty-four-hour-old F1 embryos were dechorionated, anaesthetized with 160 mg/L tricaine, and mounted in 1% low melting agarose in E3. Imaging was carried out using a Zeiss LSM800 confocal microscope with a 10× and a 40× water immersion objective.

### Spontaneous movement and light stimulation

*GAL4s1020t, UAS:ACR1-eYFP* and *GAL4s1020t, UAS:ACR2-eYFP* embryos were screened with a fluorescence stereomicroscope at 23–24 h post-fertilization to identify ACR-expressing fish. Embryos, still within their chorions, were then placed in a glass dish with 24 concave wells on a stereomicroscope (Zeiss Stemi 2000) with a transmitted light base. Behaviour was recorded on the microscope using a Point Gray Flea2 camera controlled by MicroManager. Stimulating light was delivered by LED backlights (TMS Lite), with peak intensity at 470 nm (blue), 525 nm (green) or 630 nm (red), placed adjacent to the glass dish. The power used for high intensity illumination was the maximum that could be delivered by these LEDs. The same voltage settings were used with the three light boxes to give high, medium and low intensities, but the irradiance produced differed. We provided 595-nm (amber) illumination using LEDs from CREE (XR7090-AM-L1-0001), which were mounted onto thermal LED holders (803122; Bergquist Company). The intensity of light was measured using an S120VC power sensor and a PM100A console (Thorlabs, Newton, NJ, USA). LEDs were switched on and off using an Arduino board controlled by MicroManager to regulate the power supply unit. Embryos were recorded for a total of 45 s, with the LED being turned on 15 s after the start of recording and turned off 15 s later. Each embryo was tested once for each condition and tested with different intensities and wavelengths.

### Analysis of behaviour recordings

Image analysis was carried out using Fiji (RRID:SCR_002285) [28] as well as scripts written in Python. From the raw recordings, one frame was extracted per second to obtain a total of 46 frames (including the first and last frames). Circular regions of interest (ROIs) were manually drawn around each chorion to isolate each fish. Each frame was then subtracted from the next frame to identify the differences between frames. The number of different pixels in each ROI was taken as a measure of movement of each embryo [22]. Any embryo that did not move during the entire recording was discarded from analysis. Estimation statistical methods were employed to analyse mean differences between control and experimental groups [29–31]. The 95% confidence intervals (CIs) for the mean difference were calculated using bootstrap methods [32]. All CIs were bias-corrected and accelerated [33], with resampling performed 5000 times. All reported *P* values are the results of Wilcoxon *t* tests.

### Two-photon calcium imaging

Triple transgenic zebrafish embryos (*Et(-0.6hsp70l:Gal4-VP16)s1020t, UAS:ACR1-eYFP, elavl3:GCaMP6f*) were mounted in agarose (2% low melting temperature, in E3). To enable individual cells to be followed without motion artefacts, fish were anesthetized with mivacurium chloride (Mivacron; GSK, Auckland, New Zealand). All imaging was performed using an upright Nikon A1RMP two-photon microscope equipped with a 25× 1.1 NA water immersion objective. Images were captured at a rate of 1 Hz, with the laser tuned to 920 nm. Blue light was delivered with the same light box used for behaviour experiments, at the maximum intensity. Green light was not used, as this overlaps with the emission spectrum of GCaMP6f and would saturate the detector.

### Analysis of calcium imaging data

Analysis was carried out using Fiji [28], unless otherwise stated. Background correction was first performed by subtracting the average value of a region outside the embryo for each frame. This was done to eliminate the bleed-through from the illuminating LED. A median filter with a radius of 1 pixel was then applied. Images were registered using TurboReg in Fiji [34]. ROIs were drawn manually around cells. The average fluorescence intensity of cells within an ROI was obtained by measuring only pixels above a threshold, so that pixels without a signal, but that were located within the ROI, did not reduce the value.

### Acridine orange label

Embryos were incubated in acridine orange (Sigma A6014) at a concentration of 0.01 mg/ml for 30 min, then rinsed three times in E3. They were then anesthetized in buffered MS222, mounted in 2% agarose in E3 and imaged with a 10× water immersion objective on an LSM 800 laser scanning confocal microscope (Zeiss) at 1024 × 1024 resolution. The number of labelled nuclei in the nervous system was counted manually with the aid of the multipoint tool in Fiji.

## Results

### Transgenic zebrafish express GtACR1 and GtACR2 in neurons

**Fig. 1:**
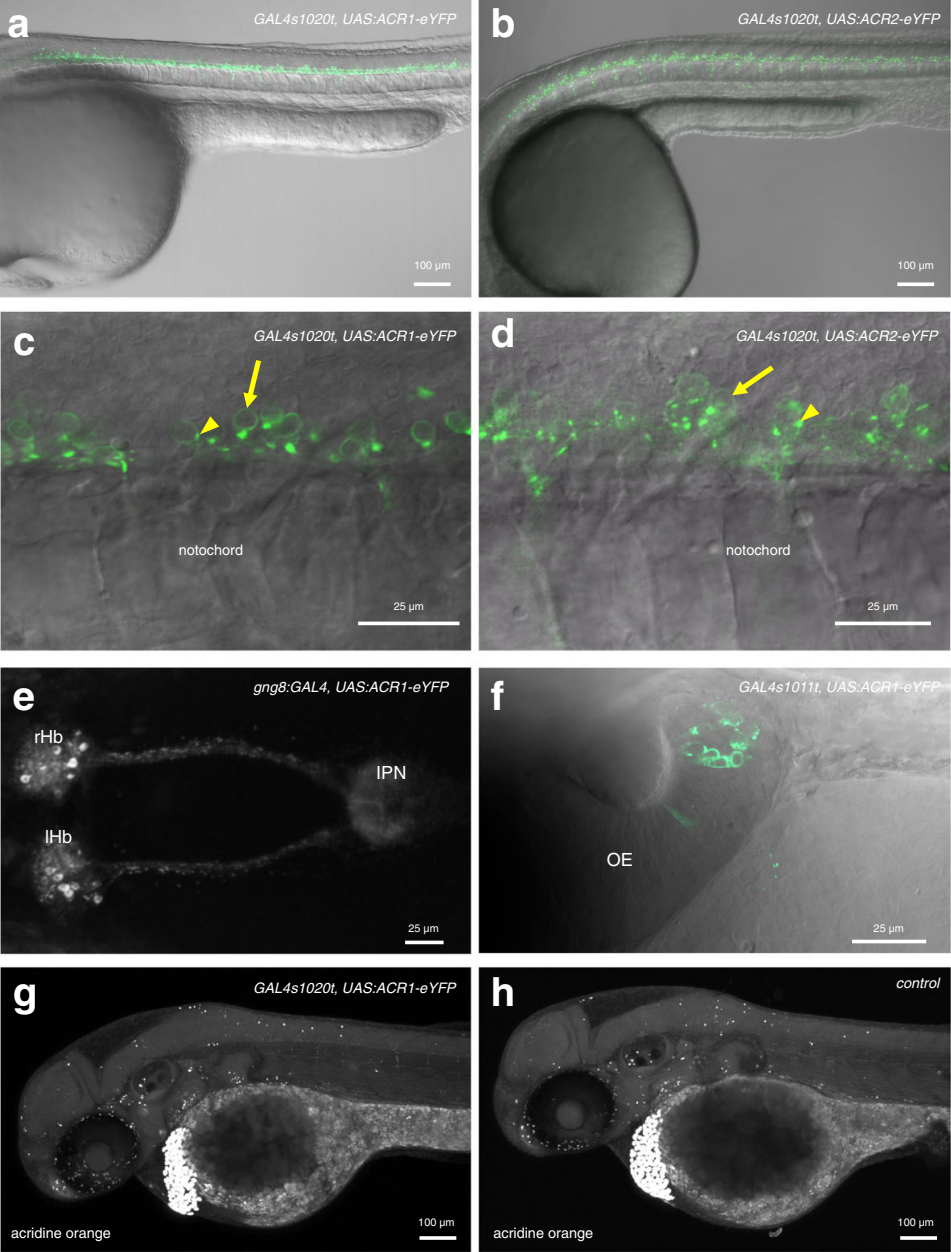
Expression of GtACR1 and GtACR2 in transgenic zebrafish. **a**, **b** Expression of GtACR1-eYFP in the trunk of 1-day-old *GAL4s1020t, UAS:GtACR1-eYFP* (**a**) and *GAL4s1020t, UAS:GtACR2-eYFP* (**b**) embryos. Labelled cells are located in the ventral regions of the spinal cord. **c**, **d** High magnification view of the trunk of fish expressing GtACR1-eYFP (**c**) or GtACR2-eYFP (**d**) in spinal neurons. There is label in the plasma membrane (*arrows*). For GtACR2, the label is dimmer. Puncta are visible (*arrowheads*). **e** An 8-dpf *gng8:GAL4, UAS:GtACR1-eYFP* larva, showing label in habenula neurons and in axons innervating the interpeduncular nucleus. **f** Expression of GtACR1-eYFP in olfactory neurons of a 3-day-old fish carrying the GAL4s1011t driver. **g**, **h** Forty-eight-hour-old embryos labelled with acridine orange. Dying cells are strongly labelled and appear as bright spots. These are detected in both GtACR1-expressing (**g**) and non-expressing (**h**) fish. There is no evidence of cell death in the ventral spinal cord of GtACR1-expressing fish. Anterior is to the left in all panels except **f**, where anterior is to the top. All panels are lateral views, except **e** and **f**, which are dorsal views. Panels **a**, **c**, **d** and **f** are single planes, while **b**, **e**, **g** and **h** are maximum projections. *OE* olfactory epithelium, *IPN* interpeduncular nucleus, *lHb* left habenula, *rHb* right habenula

To express the anion channelrhodopsins in zebrafish, transgenic lines containing the coding sequences under the control of the UAS were generated using Tol2-mediated transgenesis. When crossed with GAL4 drivers, expression of the protein could be detected by the eYFP tag (Fig. 1a–f). GtACR1 and GtACR2 were detected in the cell membrane (Fig. 1c, d). Puncta could be detected with both channels (arrowheads in Fig. 1c, d), although membrane labelling with GtACR2-eYFP was less prominent (Fig. 1d), suggesting that GtACR2-eYFP was not efficiently trafficked to the plasma membrane. When placed under different drivers, GtACR1-eYFP and GtACR2-eYFP could be detected in different regions of the nervous system, including the spinal cord, habenula and olfactory epithelium (Fig. 1c–f). Expression could be detected at various stages, ranging from early development (Fig. 1a–d) to 8 days post-fertilization (dpf) (Fig. 1e; see also Fig. 8 in the companion manuscript [35]). Transgenic fish expressing GtACR1 or GtACR2 could be grown to adulthood (>100 fish each) and could generate viable offspring. When embryos were incubated in acridine orange, a marker of dying cells [36], no dying cells were detected in regions expressing the transgene (Fig. 1 g, compare with Fig. 1 h). Scattered label was seen elsewhere, and the mean number of acridine-orange labelled cells in the nervous system of *GAL4s1020t, UAS:GtACR1* fish (*n* = 5) was 34.6 [95% CI 31.5 37.7], while the mean in non-expressing siblings (*n* = 5) was 37.2 [95% CI 31.8 42.6]. These values are not substantially different (mean difference = –2.6 cells [95% CI –8.4, 2.6]), suggesting that GtACRs can be expressed in zebrafish neurons without inducing cell death.

### Light-actuated GtACR1 and GtACR2 inhibit spontaneous movement

**Fig. 2:**
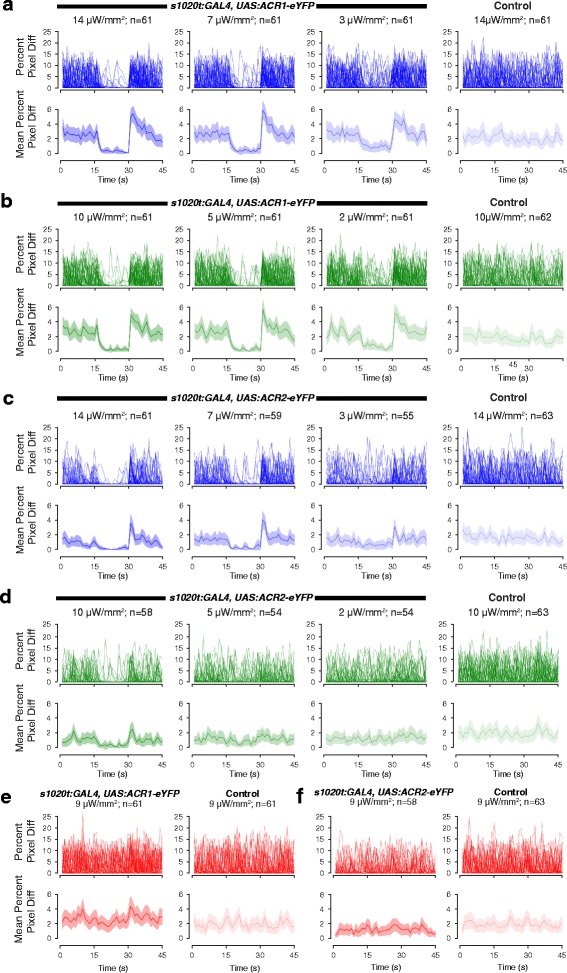
Light sensitivity of GtACR1- and GtACR2-expressing fish. **a**, **b** Response of ACR1 fish to blue (**a**) and green (**b**) light of three different intensities (high, medium and low). For each panel, the top trace shows the movement of each fish in 1 s. This is measured by number of pixels that differ, relative to the cross-sectional area of the chorion. Each line represents one fish. The lower trace shows the mean value and 95% CIs. Controls refer to siblings lacking eYFP expression that were exposed to high intensity light. **c**, **d** Effect of blue (**c**) and green (**d**) light of different intensities on ACR2-expressing fish. **e**, **f** The effect of red light on ACR1- (**e**) and ACR2- (**f**) expressing fish. Line colour corresponds to the colour of light used for illumination. For all conditions, the period of illumination lasted from *t* = 15 s to *t* = 30 s

To assess the ability of GtACR1 and GtACR2 to inhibit neural activity, we used spontaneous movement as an assay. Between 17 and 27 h post-fertilization, zebrafish display spontaneous coiling movements [37] that are dependent on the activity of spinal neurons [38]. As the rate of coiling varies strongly with age, non-expressing siblings were used as controls. We tested the effect of different intensities of light of different wavelengths (Fig. 2) on spontaneous movement of fish expressing these channels in spinal neurons under the control of the 1020 GAL4 driver. Fish were exposed to a 15-s pulse of light in the middle of a recording lasting 45 s. The amount of movement per animal was measured in terms of pixel value differences between subsequent frames.

**Fig. 3:**
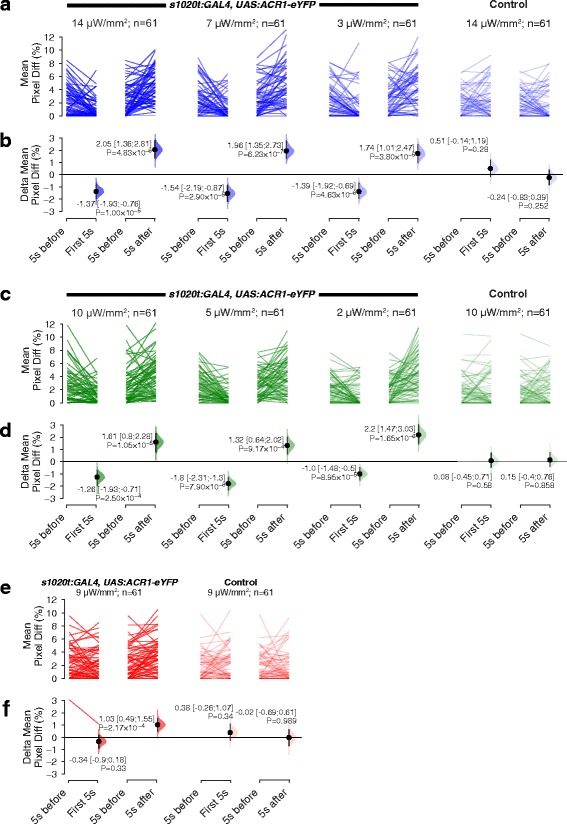
The effect of light and loss of light on spontaneous movement of GtACR1 embryos. **a**, **c**, **e** The amount of movement displayed by individual embryos, in the 5 s before light onset (‘5 s before’), in the first 5 s after light onset (‘First 5 s’) and in the first 5 s after light offset (‘5 s after’). Different intensities of blue (**a**), green (**c**) and red (**e**) light were tested. Controls refer to siblings lacking eYFP expression. The line colour of each plot corresponds to the light colour employed for illumination. **b**, **d**, **f** The mean amount of movement relative to the period before light onset. A negative value indicates inhibition of spontaneous movement, whereas a positive value indicates elevated levels of movement. Mean differences and 95% CIs are reported alongside *P* values from Wilcoxon tests. Blue (**b**) and green (**d**) light both affect spontaneous movement. Red light has a small effect (**f**)

**Fig. 4:**
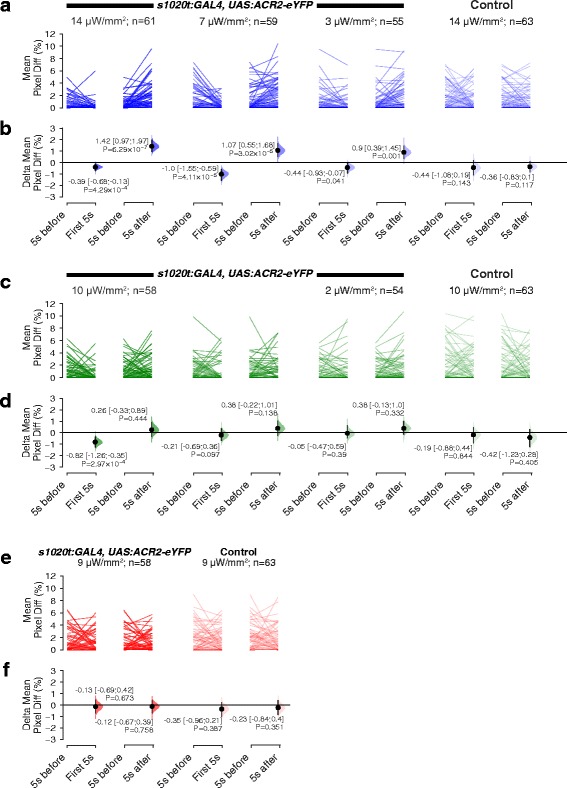
The effect of light and loss of light on spontaneous movement of GtACR2 embryos. **a**, **c**, **e** The amount of movement displayed by individual embryos, in the 5 s before light onset (‘5 s before’), in the first 5 s after light onset (‘First 5 s’) and in the first 5 s after light offset (‘5 s after’). Three different intensities of blue (**a**) and green (**c**) light and one intensity of red light (**e**) were tested. The colour of the lines indicates the colour of light used for illumination. Controls for each experiment were siblings not expressing eYFP. **b**, **d**, **f** The mean amount of movement relative to the period before light onset. A negative value indicates inhibition of spontaneous movement, whereas a positive value indicates elevated levels of movement. Mean differences and 95% CIs are reported; all *P* values are results of Wilcoxon tests. Blue (**b**) light affected spontaneous movement at medium and high intensities, whereas green light only had a weak effect at high intensity (**d**). Red light had no effect

High and medium intensities of green and blue light had a similar effect on GtACR1-expressing fish, namely inhibition of coiling (Figs. 2a, b, 3a–d; Movie 1, see Additional file 1). Siblings that did not express the channel continued to coil in the presence of high intensity light (fourth column of Fig. 2a, b; Movie 2, see Additional file 2). Low intensities (3 μW/mm^2^ for blue and 2 μW/mm^2^ for green light) were able to cause freezing, but at reduced efficiency compared to the higher intensities (compare the third column in Fig. 2a, b with the first two columns). For GtACR2-expressing fish, high and medium intensities of blue light were able to induce similar levels of freezing, while a low level of blue had a small effect (Figs. 2c, 4a, b); green light inhibited embryo movement only when used at high intensity (Figs. 2d, 4c, d). Red light did not inhibit coiling of either GtACR1 or GtACR2 fish at the intensity tested (Figs. 2e, f, 3e, f, 4e, f), consistent with the reported action spectrum of both GtACR1 and GtACR2 [21]. Together, these data suggest that GtACR1 is more effective than GtACR2.


Additional file 1: Movie 1. The effect of 10 μW/mm^2^ green light on spontaneous movement of *GAL4s1020t, UAS:GtACR1* embryos. Twenty-four-hour-old embryos exhibit spontaneous coiling, except during the period of green light delivery, which is indicated by the *green dot* on the *bottom left*. (MP4 975kb)
Additional file 2: Movie 2. The effect of 10 μW/mm^2^ green light on spontaneous movement of embryos without GtACR1 expression. Spontaneous coiling persists during delivery of green light. (MP4 921kb)


### GtACRs increase behavioural activity at light offset

When the actuating light was turned off, an increase in the movement of GtACR1- and GtACR2-expressing embryos was detected (Fig. 2a–c lower trace). To assess whether this reflected an increased amount of movement per animal, or an increased probability but similar level of movement, we looked at the response of each fish, comparing movement in the 5-s period after light offset with the 5-s period before light onset. As can be seen in Figs. 3 and 4, there is a greater amount of movement following light offset, implying that loss of light (light OFF) triggers stronger movement than what is seen spontaneously.

This effect was observed in larvae expressing GtACR1 in both green and blue light, at all three light intensities tested (Fig. 3a–d). However, for larvae expressing GtACR2, this effect was only observed upon exposure to blue light (Fig. 4a, b). This suggests that the movement to light offset is not due purely to change in illumination (i.e. a startle response), but is caused by the light-gated anion channel.

### Comparison of GtACRs with halorhodopsin

**Fig. 5:**
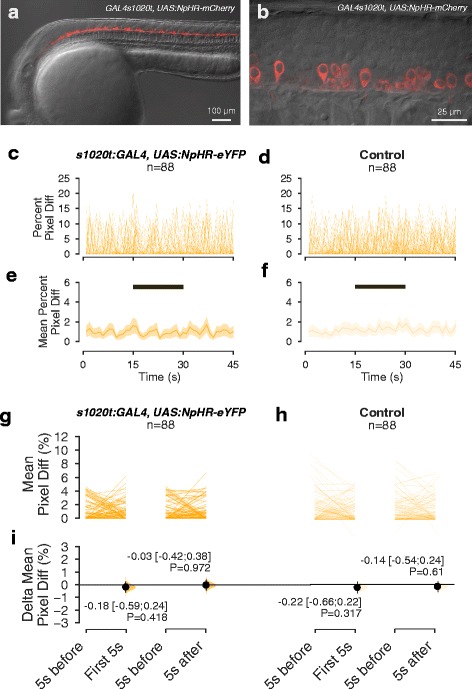
The effect of low intensity amber light on spontaneous movement of NpHR-expressing embryos. **a**, **b** Lateral view of a 24-h-old embryo expressing NpHR-mCherry under the 1020 GAL4 driver. **a** Overview of expression in the spinal cord. **b**. High magnification view, showing expression of NpHR-mCherry in spinal neurons. **c**–**f** Time course of movement of embryos, with (**c**, **e**) or without (**d**, **f**) halorhodopsin expression, in a 45-s recording with exposure to 15 s of amber light. The period of illumination is indicated by the *black bar*. **c** and **d** show movement of individual embryos, while **e** and **f** show mean values and 95% CIs. **g**, **h** Average amount of movement in the 5 s before light onset (‘5 s before’), in the first 5 s after light onset (‘First 5 s’) and in the first 5 s after light offset (‘5 s after’), in NpHR-expressing embryos (**g**) and non-expressing siblings (**h**). **i** Difference in movement in the first 5 s after light onset and after light offset, relative to the period before light, in NpHR-expressing and non-expressing embryos. Intensity of amber light used = 17 μW/mm^2^

Halorhodopsin has previously been used to inhibit neural activity in zebrafish larvae [39]. We compared the performance of this chloride pump with the anion channelrhodopsins. At the intensities at which GtACRs enabled a behavioural manipulation of zebrafish, no light-evoked inhibition of spontaneous movement or induction of movement at light offset was seen in fish expressing NpHR-mCherry (Fig. 5). This is consistent with published reports that stronger light (e.g. 19 mW/mm^2^) is required for halorhodopsin-mediated inhibition of spinal neuron activity in the *GAL4s1020t,UAS:NpHR-mCherry* line [39]. Thus, anion channel rhodopsins are more effective tools for optical control of behaviour in zebrafish, compared with halorhodopsin.

### Calcium imaging of spinal neurons

**Fig. 6:**
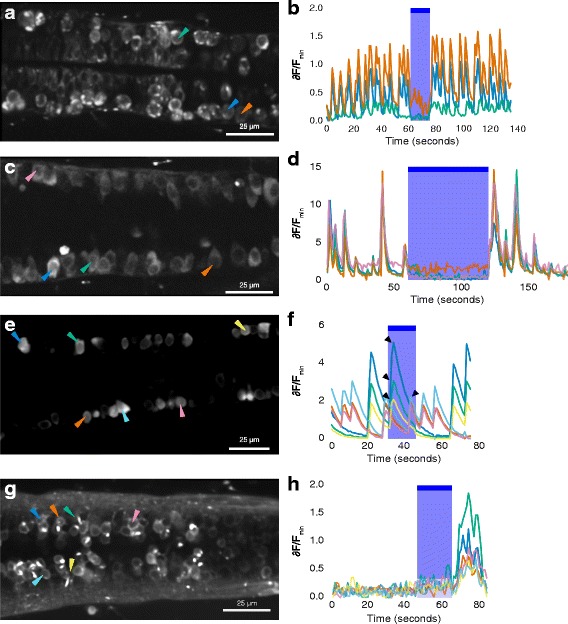
Calcium imaging of spinal neurons in GtACR1 embryos and non-expressing siblings. **a** Ventral spinal neurons of a 24-h-old *1020:GAL4, UAS:GtACR1-eYFP, elavl3:GCaMP6f* embryo. The *green arrowhead* indicates a neuron with bright membrane label and puncta, suggesting expression of GtACR1-eYFP. The *orange arrowhead* indicates a neuron without GtACR1-eYFP expression. **b** Time course of fluorescence intensity in neurons, represented as change relative to minimum fluorescence. There is a reduction in activity during delivery of blue light. **c**, **d** Activity in dorsal spinal neurons, which do not express the GAL4 driver, in another 24-h-post-fertilization (hpf) *GAL4s1020t, UAS:GtACR1-eYFP, elavl3:GCaMP6f* embryo. Spontaneous activity was detected before and after, but not during, the period of blue light delivery. **e**, **f** Spontaneous activity in six neurons in a 26-h-old embryo with no GtACR1-eYFP expression. **e** Increase in fluorescence intensity occurs during the period of illumination with blue light (*arrowheads*). **g**, **h** The response of six spinal neurons in a 4-day-old embryo expressing GtACR1. There is no activity before light in these neurons, but there is a rise in GCaMP6f fluorescence after termination of the blue light. In panels **b**, **d**, **f** and **h**, the *blue shaded region* and *bar* indicate the period in which light was delivered. The colours of the traces represent relative change in fluorescence of the cells indicated by the *arrowheads* with the corresponding colours in the image on the *left*


As an independent method of assessing the effect of light and loss of light on neurons in zebrafish larvae expressing GtACR1, two-photon calcium imaging was carried out. We used the *elavl3* promoter to drive broad neuronal expression of the calcium indicator GCaMP6f. Cells expressing GtACR1-eYFP could be identified by the presence of strong membrane fluorescence (see, e.g. the green arrowhead, Fig. 6a). Spontaneous fluctuations in GCaMP6f fluorescence could be detected in these cells, and this was reduced in the presence of blue light (Fig. 6b). Cells without a bright membrane label (e.g. the orange arrowhead in Fig. 6a) also showed a loss of spontaneous calcium transients in the presence of actuating light, consistent with loss of activity in the spinal network, and not only in GtACR1-expressing cells. To further explore this, imaging was carried out at a more dorsal plane of *elavl3:GCaMP6f, GAL4s1020t, UAS:GtACR1* fish, where cells do not express GtACR1-eYFP (Fig. 6c; see also Fig. 1). Here, again spontaneous calcium spikes were absent in the presence of blue light (Fig. 6d; Movie 3, see Additional file 3). Suppression of calcium transients was seen over the entire period in which light was delivered, which was 60 s, suggesting that inhibition of activity can occur for longer than the 15 s used in the behaviour test. Additionally, this indicates that, at the offset of blue light, fluctuations in calcium levels resumed. In siblings that did not express GtACR1, fluctuations in calcium levels persisted in the presence of light (Fig. 6e, f), indicating that light itself cannot suppress calcium transients in these fish.

Additional file 3: Movie 3. The effects of blue light on the trunk of *GAL4s1020t*, *UAS:GtACR1*, *elavl3:GCaMP6f* fish. Twitches of the body are accompanied by increase in GCaMP6f fluorescence. In the presence of blue light (from 60 to 120 s), which can be seen by an overall increase in brightness, the embryo no longer twitches and there is no fluctuation in GCaMP6f fluorescence. After the blue light is switched off, movement and change in GCaMP6f fluorescence resume. This is a dorsal view, with anterior to the *left*. (AVI 2660kb)


To test whether offset of the actuating light can drive neural firing in fish expressing GtACR1, as suggested by behavioural data, we used fish at a later stage where spontaneous activity was not as prominent, so that signals evoked by loss of light can be clearly distinguished from spontaneous activity. As seen in Fig. 6 g, h, spinal neurons that had no activity before light showed an increase in fluorescence after the offset of blue light. This observation suggests that the termination of light-gated silencing mediated by GtACR1 can lead to depolarization of neurons within the spinal network.

## Discussion

We have investigated the usefulness of anion channelrhodopsins from *Guillardia theta* as a tool for optical control of larval zebrafish behaviour. By expressing these channels in spinal neurons of larval zebrafish and exposing the animals to light, spontaneous coiling movements could be completely and reversibly inhibited. GtACR1 appears to be a more effective tool, as ACR1-expressing fish were affected by both green and blue light at the lowest intensity tested, which is ~3 μW/mm^2^. GtACR2 was able to inhibit movement, but it was effective mainly with blue light at medium or high intensity; green light could inhibit movement only at high intensity. This is similar to findings in *Drosophila*, where 1.3 μW/mm^2^ of green light was sufficient to actuate GtACR1, whereas a higher intensity of blue or green light was required for GtACR2 [22]. Halorhodopsin, which has been shown to be effective in *GAL4s1020t* zebrafish when actuated with amber light at ~20 mW/mm^2^ [39], appeared to be unaffected by the low intensity that could be used with GtACR-expressing embryos. This suggests that, as in *Drosophila* [22], the anion channelrhodopsins are potent tools for light-mediated reversible inhibition of neural activity in zebrafish.

The assay adopted here to establish parameters for use of GtACR1 and GtACR2 in zebrafish larvae is spontaneous coiling. Friedman et al. have found that dark-adapted AB wild-type larvae, which coil in the dark, stop coiling when exposed to light due to the presence of extraretinal opsins [40]. We find no light-evoked inhibition of coiling in control fish that lacked GtACRs in our experiments. The reason for this difference is unclear. Regardless of the cause, this observation reflects an important factor that should be taken into consideration when designing optogenetic experiments with larval zebrafish, namely the presence of extraretinal opsins. The zebrafish has at least 42 opsins [41], many of which are expressed outside the retina [42, 43] and can affect behaviour independently of the visual system [44, 45]. Innate behaviours can be triggered by low levels of light [46] — within the range that actuates GtACRs. Thus, when performing optogenetic manipulations, an essential control is the use of siblings that do not express the channel that is being used to manipulate the cells of interest.

Although the GtACRs are potent tools, there may be some limitations that should be borne in mind. Anion channelrhodopsins may not be able to silence neural activity in all cells. As noted by Wiegert et al. [47], actuating these pumps in cells with high levels of intracellular chloride may lead to depolarization. Thus, characterization of cellular response should be undertaken to confirm that there is loss of activity. Additionally, these pumps may be useful only for short-term inhibition, in the range of seconds and possibly up to 1 min (see the companion manuscript [35] and Fig. 6c, d) [47, 48]. It is unclear whether they can be chronically actuated. Although the pumps are sensitive, it does not seem that expression during larval development and growth has strong adverse effects. We do not find evidence that expression of the pumps causes cell death, and larvae expressing GtACR1 or GtACR2 can grow to adulthood and give rise to viable offspring. Nevertheless, to control for potential developmental effects, the behaviour of siblings that do not express the transgene can be compared to that of expressing siblings, in the absence of the actuating light. The use of GtACR2, which is less sensitive than GtACR1, may also minimize potential adverse effects from ambient light. Finally, it should be noted that the termination of light-evoked silencing can lead to depolarization of neurons. This could be seen in larval zebrafish where GtACRs were expressed in spinal motor neurons, as judged by increased coiling behaviour as well as a rise in intracellular calcium of spinal neurons at the offset of light. This property of GtACRs has been observed in other light-gated chloride pumps, including halorhodopsin [17], and it is linked to an increase in excitability due to accumulation of chloride ions inside the cell, which elevates mean spike probability and mean stimulus-evoked spike rate by changing the reversal potential of the GABA_A_ receptor [49]. Thus, in designing experiments where the goal is to test the effects of silencing a particular set of neurons, it would be advisable to restrict observations to the period during which light is delivered. The burst of activity that occurs after the offset of light may be problematic in a study of long-term processes, such as memory [50] or emotion [51]. For acute processes, however, this property may be beneficial, as it provides a way to test the effects of activating the same set of neurons. An example of this is shown in the companion manuscript [35], where the direction of swimming is reversed in light versus darkness when GtACRs are expressed in the anterior thalamus.

## Conclusions

The anion channelrhodopsins GtACR1 and GtACR2 enable optical inhibition of neural circuits in zebrafish. They are effective at illumination levels that fail to actuate NpHR, and may thus enable efficient testing of the necessity of neurons in a given behaviour.
